# Thyroid Dysfunction in the Elderly with Hearth Failure

**Published:** 2017-04

**Authors:** Gordana MIHAJLOVIC, Miljanka VUKSANOVIC, Nebojsa DESPOTOVIC, Maja NIKOLIC-DESPOTOVIC, Predrag ERCEG, Dragoslav P. MILOSEVIC, Tomislav JOVANOVIC

**Affiliations:** 1. Faculty of Medicine, University of Belgrade, Belgrade, Serbia; 2. Clinic of Internal Diseases, Clinical Hospital Center Zvezdara, Belgrade, Serbia

## Dear Editor-in-Chief

Today there is a vast number of controversies regarding the relevance and influence of thyroid function on heart failure (HF) and in particular regarding the relevance of hypothyroidism for heart failure for the elderly (1). The prevalence of heart failure and hypothyroidism are growing in the elderly population, geriatrics and geriatricians would have something new to add to the debate (2). Aging as a risk factor for heart failure and hypothyroidism are not sufficient to explain the occurrence of the pathophysiology of these disorders (3). The aim of this study was to investigate the New York Heart Association (NYHA) classification, thyroid status (measuring free triiodothyronine (fT3), free thyroxine (fT4), Thyroid-stimulating hormone (TSH)) and possible changes regarding medicament treatment (furosemide, angiotensin-converting-enzyme (ACE) inhibitors, beta blockers (BB), spironolactone and digoxin) in hospitalized elderly (≥65 yr) patients with HF.

This matched case-control study was conducted from 2010 to 2015 at Clinical Hospital Center “Zvezdara”, in Belgrade, Serbia and was ethically approved and informed consent was taken from participants.

Demographic and anthropometric variables such as age, including NYHA class, were taken. Blood samples were assayed for serum fT3, fT4 and thyroid stimulating hormone (TSH). In addition, we evaluated the heart failure medicament therapy and the parameters of medicament therapy were addressed to six groups of drugs: diuretics (spironolactone, furosemide), ACE inhibitors, beta-blockers, cardiotonic glycosides (digoxin) and coumarin preparations (4). The significance of difference was evaluated using chi-square test, (one-way) ANOVA or Kruskal-Wallis test depending on the type of parameter analyzed. In case of registered statistical significance, the correlation was done additionally.

**Fig. 1:**
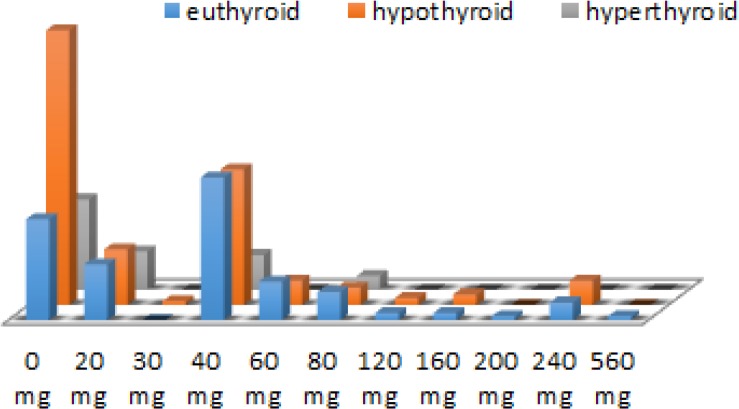
Frequences of doses of intravously applied furosemid in hospitalized elderly patients depending on thyroid state

**Fig. 2:**
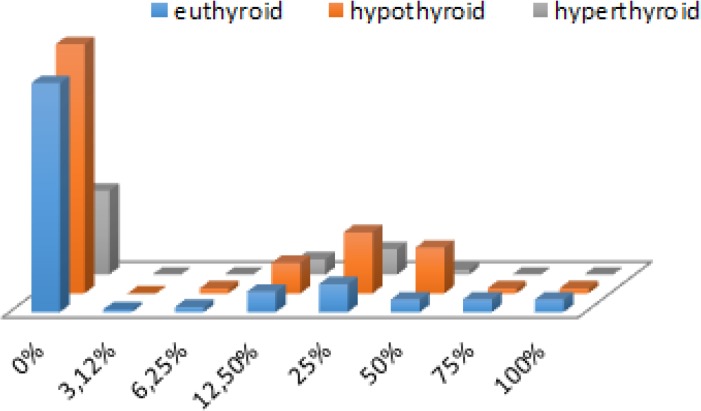
Total dose of beta-blockers in hospitalized elderly patients depending on thyroid status

Among 326 hospitalized elderly patients with HF (average aged 77.2 ± 5.9 yr), we investigated three groups of patients: with hypothyroidism (48.5%), euthyroid state (35.9%) or hyperthyroidism (5.6%). Decompensated form of HF (NYHA III or IV) was significantly more frequently registered in euthyroid group of hospitalized elderly patients than in hypothyroid or hyperthyroid patients (82.9% vs. 60.7% vs. 52.9%, *P*<0.001). According to TSH values, decompensated HF was the most common among the hospitalized elderly patients with hypothyroidism, comparing to patients with euthyroid state or with hyperthyroidism (71.3% vs. 67.6% vs. 57.1%, p=0.322) (5, 6). We found significant differences among investigated groups depending on their thyroid status on furosemide (*P*=0.002) and BB (*P*=0.025) treatment (Fig. 1). Elderly HF patients with hypothyroidism, comparing to those with euthyroid status had significantly; lower intravenously dosage of furosemide (*P*=0.014), a fewer number of days for intravenously applied furosemide (*P*=0.011), higher total doses of ACE inhibitors (*P*=0.031) and BB (*P*=0.001) (Fig. 2).

The level of TSH is a parameter closely linked to cardiovascular diseases and, more sensitive indicator of cardiovascular risk than the fT4. Besides, a significant difference among HF medicament treatment in hospitalized elderly subjects according to their thyroid status. We can emphasize the need for a specific geriatric assessment together with a systematic screening for thyroid dysfunction in all patients with 65 yr.
